# Advances in Oxygen Evolution Reaction Electrocatalysts via Direct Oxygen–Oxygen Radical Coupling Pathway

**DOI:** 10.1002/adma.202416362

**Published:** 2025-01-15

**Authors:** Chengli Rong, Xinyi Huang, Hamidreza Arandiyan, Zongping Shao, Yuan Wang, Yuan Chen

**Affiliations:** ^1^ School of Chemical and Biomolecular Engineering The University of Sydney Darlington New South Wales 2006 Australia; ^2^ Centre for Advanced Materials and Industrial Chemistry (CAMIC) School of Science RMIT University Melbourne VIC 3000 Australia; ^3^ WA School of Mines: Minerals Energy and Chemical Engineering Curtin University Perth WA 6845 Australia; ^4^ Department of Chemical Engineering The University of Melbourne Parkville VIC 3010 Australia

**Keywords:** electrocatalyst, operando characterization, oxide path mechanism, oxygen evolution reaction, oxygen–oxygen coupling

## Abstract

Oxygen evolution reaction (OER) is a cornerstone of various electrochemical energy conversion and storage systems, including water splitting, CO_2_/N_2_ reduction, reversible fuel cells, and rechargeable metal‐air batteries. OER typically proceeds through three primary mechanisms: adsorbate evolution mechanism (AEM), lattice oxygen oxidation mechanism (LOM), and oxide path mechanism (OPM). Unlike AEM and LOM, the OPM proceeds via direct oxygen–oxygen radical coupling that can bypass linear scaling relationships of reaction intermediates in AEM and avoid catalyst structural collapse in LOM, thereby enabling enhanced catalytic activity and stability. Despite its unique advantage, electrocatalysts that can drive OER via OPM remain nascent and are increasingly recognized as critical. This review discusses recent advances in OPM‐based OER electrocatalysts. It starts by analyzing three reaction mechanisms that guide the design of electrocatalysts. Then, several types of novel materials, including atomic ensembles, metal oxides, perovskite oxides, and molecular complexes, are highlighted. Afterward, *operando* characterization techniques used to monitor the dynamic evolution of active sites and reaction intermediates are examined. The review concludes by discussing several research directions to advance OPM‐based OER electrocatalysts toward practical applications.

## Graphical abstract

The development of oxygen evolution reaction (OER) electrocatalysts via the oxide path mechanism (OPM) is systematically reviewed. It sheds light on the rational design of OPM‐based OER electrocatalysts to break the activity‐stability trade‐offs involved in conventional OER mechanisms, leading to more efficient energy conversion and storage processes, such as water electrolysis, CO_2_/N_2_ reduction, reversible fuel cells, and rechargeable metal‐air batteries.

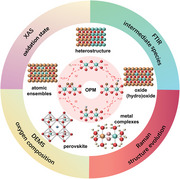

## 1 Introduction

Oxygen evolution reaction (OER) is essential in renewable energy conversion systems, such as electrochemical water splitting, CO_2_/N_2_ reduction, reversible fuel cells, and rechargeable metal‐air batteries.^[^ ^1^, ^2^, ^3^, ^4^, ^5^, ^6^ ^]^ However, OER is a kinetically sluggish reaction involving four‐electron transfers, resulting in high overpotential with significant energy inputs; thus, OER is a major bottleneck to improving the efficiency of these renewable energy systems.^[^ ^6^, ^7^, ^8^, ^9^ ^]^ OER's slow reaction kinetics stem from multiple intermediate species and high energy barriers associated with all electron transfer steps. Accelerating electron transfers between reaction intermediates and catalysts is essential to improve catalytic performance.

Three reaction mechanisms have been proposed for OER: adsorbate evolution mechanism (AEM), lattice oxygen oxidation mechanism (LOM), and oxide path mechanism (OPM).^[^ ^10^ ^]^ AEM involves a sequence of concerted electron‐proton transfer steps between catalytically active sites on electrocatalysts and oxygen intermediates, involving OH*, O*, OOH*, and O_2_.^[^ ^11^ ^]^ There are linear scaling relationships in the adsorption energy of these oxygen intermediates, which limits the improvement of electrocatalysts’ catalytic activity. In contrast, LOM suggests that OER proceeds via the direct generation of OH^*^, O_2_^2−^, and O_2_.^[^ ^12^ ^]^ This reaction pathway is kinetically more favorable as it bypasses the formation of OOH*, thus potentially circumventing high overpotentials required in AEM. However, defects in electrocatalysts, such as oxygen vacancies, are needed in LOM, which would extensively disrupt catalysts’ crystal structures—causing severe dissolution of active sites, which leads to unstable reaction performance. Unlike AME and LOM, OPM suggests that OER proceeds via direct oxygen–oxygen (O─O) radical coupling without creating defects or generating additional reaction intermediates (OOH*). It would only involve O* and OH* as reaction intermediates.^[^ ^13^ ^]^ Consequently, the trade‐off between catalytic activity and stability encountered by AEM and LOM does not exist in LOM, creating the hope of having highly active and stable OER electrocatalysts.

**Scheme 1:**
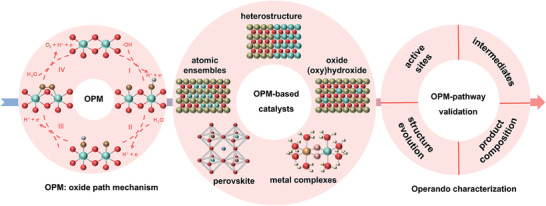
Schematic of the main content for OPM‐based OER electrocatalysts discussed in this review.

Significant recent efforts have been made to develop electrocatalysts that can drive OER along the OPM pathway.^[^ ^13^, ^14^, ^15^, ^16^, ^17^, ^18^, ^19^ ^]^ However, challenges persist because there is still a lack of comprehensive understanding of these electrocatalysts’ structure and their structural evolution at the atomic scale during OER, which prevents more efficient catalyst designs. This review summarizes recent advances in developing OPM‐based OER electrocatalysts, beginning with an analysis of their reaction mechanisms to guide understanding of electrocatalyst design strategies. We further discuss recently reported electrocatalysts, such as atomic ensembles, metal oxides, metal oxyhydroxides, perovskite oxides, and molecular complexes. We highlight their new design concepts to drive OER via the OPM pathway. Next, the roles of *operando* characterization in tracking dynamic evolutions of active sites and reaction intermediates to validate OPM are discussed. Finally, the review proposes future research directions to realize OPM‐based OER electrocatalysts toward practical applications (**Scheme** **1**).

## 2 OER Mechanisms

Understanding OER mechanisms is crucial for the rational design of effective OER electrocatalysts. As described earlier, three OER mechanisms have been proposed, and they are strongly correlated with electrocatalysts’ geometric and electronic structures.

### 2.1 Adsorbate Evolution Mechanism

**Figure 1:**
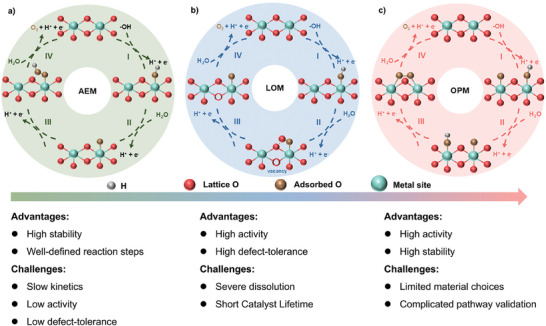
Schematic of potential OER pathways according to a) AEM, b) LOM, and c) OPM pathway, and their proposed advantages and disadvantages.

AEM suggests that OER would proceed in four steps. Each step involves a proton‐coupled electron transfer over metal active sites on catalyst surfaces (**Figure** **1**a).

Initially, oxygenated intermediates, such as OH^−^ or H_2_O, adsorb onto active sites, forming OH*. This step is followed by the deprotonation of OH* to generate O* species. Subsequently, O* species undergo nucleophilic attack by OH^−^ or H_2_O to form OOH*, which finally evolve into O_2_ molecules and detach from the active sites. Koper et al. proposed a general scaling relationship between the binding energies of OH* and OOH* on active sites with a constant energy difference of 3.2 ± 0.2 eV (i.e., ∆*G*_OOH*_−∆*G*_OH*_).^[^ ^20^ ^]^ This scaling relationship is critical as either ∆*G*_OH*_ or ∆*G*_OOH*_ can act as the rate‐determining step (RDS) in OER. The O* species in the middle of this scaling relationship are crucial, and the value of Δ*G*_O*_ − Δ*G*_OH*_ has been commonly used to predict an electrocatalyst's catalytic activity. Montoya et al. have illustrated this correlation between Δ*G*_O*_ − Δ*G*_OH*_ and catalytic activity using a volcano plot for metal oxide‐based electrocatalysts, indicating a minimum theoretical overpotential of 370 mV via the AEM pathway.^[^ ^21^ ^]^ These suggest that the catalytic activity of electrocatalysts is fundamentally limited in AEM. Breaking the linear scaling relationship is required to achieve better catalytic performance. This has been explored by introducing multi‐component catalysts with tunable active sites that allow independent optimization of the binding energy of different oxygen species. Modifying electronic structures of active sites through alloying or creating heterostructures could further enhance catalytic activity. In addition, the development of proper catalyst surface treatments to prevent poisoning active sites while maintaining the accessibility of active sites is crucial.

### 2.2 Lattice Oxygen‐Evolution Mechanism

According to LOM (Figure 1b), the first two reaction steps involving the formation of O* and OH* species are similar to those in AEM. However, LOM suggests that surface O* species would combine with lattice oxygen in electrocatalysts, resulting in direct O─O bond formation. This process continues as the resulting surface vacancies are refilled by H_2_O molecules in electrolytes, which form OH* species, accompanied by the release of a proton through a one‐electron oxidation step.^[^ ^22^ ^]^ OER proceeding via the LOM pathway would have a significant advantage by circumventing the overpotential constraint in the AEM pathway because the formation of OOH* is bypassed.^[^ ^12^ ^]^ Besides, catalytic active sites in LOM are not limited to metal sites; lattice oxygen also participates in the reaction and serves as additional active sites.

Nevertheless, the durability of metal oxides is a critical issue in LOM due to the bulk oxygen diffusion and structural reconstruction caused by the continuous formation of oxygen vacancies and dissolution of cations during lattice oxygen redox. This explains why electrochemically synthesized (defect‐rich) RuO_2_ shows a higher dissolution rate than its thermally prepared counterpart.^[^ ^23^ ^]^ Besides, generating defects would expose metal atoms to the catalyst–electrolyte interface, which can be overoxidized to high valence states with more solubility. In addition, creating oxygen vacancies alters catalysts’ electronic structure, potentially weakening metal–oxygen bonds. This can exacerbate metal dissolution, eroding the catalyst's active sites and reducing long‐term performance. In other words, OER activity enhancement obtained via LOM often comes at the expense of poor long‐term thermodynamic stability of electrocatalysts. Besides, whether lattice oxygen in electrocatalysts in LOM should be entirely and/or partially inhibited or activated to improve catalyst performance is still under debate.

Accordingly, stabilizing lattice structures without inhibiting oxygen mobility is critical for advancing catalysts based on the LOM pathway. This may be achieved by designing catalysts with robust frameworks that resist lattice destabilization. In addition, incorporating protective coatings or doping with inert elements can mitigate structural degradation. Advanced computational tools can aid in designing such catalysts with optimal oxygen transport properties while maintaining their structural integrity.

### 2.3 Oxide Path Mechanism

OPM involves a compelling reaction pathway surpassing the theoretical limits of OER activity and stability in AEM and LOM. OPM leverages two metal sites with optimal atomic distance to facilitate direct O─O radical coupling, whereby only O* and OH* are involved as OER intermediates, leading to O_2_ release without additional protons or electrons forming OOH* (Figure 1c). Therefore, OER, according to OPM, could efficiently break the linear scaling relationship in AEM, resulting in lower overpotential and higher overall energy efficiency. In addition, lattice oxygen would remain intact in OPM, and OER occurs solely through surface‐bound active sites. This eliminates the need for lattice oxygen vacancy formation, alleviating electrocatalysts’ structural collapse and ensuring long‐term stability under harsh OER conditions.

OER via OPM involves direct O─O radical coupling opens the possibility of electrocatalysts with both high activity and stability. However, OPM imposes more stringent requirements on the active sites’ geometric and electronic configuration. To drive OER via the OPM reaction pathway necessities precise controls over the atomic distance between adjacent active sites and their electronic properties to promote effective O─O radical coupling.

## 3 OPM‐Based OER Catalysts

New OPM‐based OER electrocatalysts have been developed across five material categories: 1) atomic ensembles, 2) metal oxides with heterostructures engineered by element doping, 3) metal oxyhydroxides, 4) perovskite oxides, and 5) metal complexes. The following section highlights recent advancements in developing electrocatalysts in these materials categories. Several general synthesis strategies exist for all materials, such as regulating the distance between active sites, controlling the coverage of reaction intermediates, and optimizing catalysts’ oxidation states.

### 3.1 Atomic Ensembles

Single‐atom catalysts (SACs) contain isolated metal atoms as active sites dispersed on a support matrix and have been intensively investigated for various energy conversion and storage systems.^[^ ^24^, ^25^, ^26^, ^27^, ^28^, ^29^, ^30^, ^31^, ^32^ ^]^ They offer distinct advantages compared to other metal nanoparticle‐based catalysts, such as fully exposed active sites with tunable local coordination environments, which enhance their catalytic performance.^[^ ^33^ ^]^ However, individual active sites in SACs often cannot break the linear scaling relationships suggested by AEM.^[^ ^34^ ^]^ Besides, chemical bonds between single metal atoms and support matrixes are generally weak, making them highly susceptible to corrosion or dissolution.^[^ ^35^, ^36^ ^]^ This vulnerability can be further aggravated by the increased involvement of lattice oxygen in support matrixes, resulting in fast deactivation under harsh OER conditions. The coordination environment of active sites in catalysts, either single atoms, ensembles of two or several sites regularly arranged, or metal clusters, strongly impacts their catalytic activities.^[^ ^13^, ^16^, ^37^ ^]^ Regulating active sites’ coordination environments in SACs shows a great potential to change reaction pathways, leading to improved catalytic activity and stability.^[^ ^15^, ^38^, ^39^ ^]^

**Figure 2:**
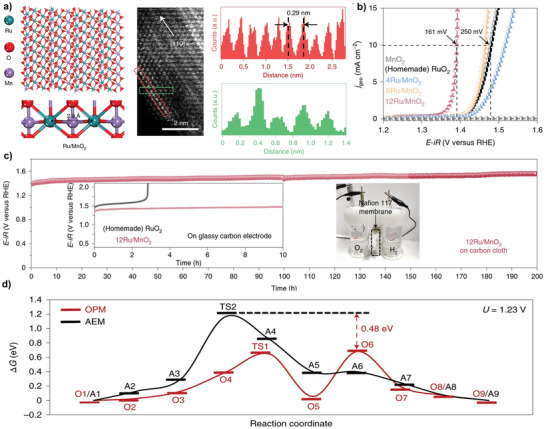
a) Crystalline structure model of α‐MnO_2_ with Ru substituting at surface sites (left), an aberration‐corrected HAADF‐STEM image (middle), and line profiles (right) of 12Ru/MnO_2_. b) Polarization curves and c) Chronopotentiometry results of 12Ru/MnO_2_ with reference samples in 0.1 m HClO_4_ electrolyte. d) The free energy (Δ*G*) diagrams of reaction pathways suggested by AEM and OPM on 12Ru/MnO_2_ at 1.23 V. Reprinted with permission.^[^ ^13^ ^]^ Copyright 2021, Nature Publishing Group.

For example, Lin et al. reported a patch of single Ru atom arrays supported by crystalline α‐MnO_2_ nanofibers as highly active and acid‐stable OER electrocatalysts.^[^ ^13^ ^]^ During the catalyst synthesis, a cation exchange reaction allowed the substitution of Ru atoms for Mn atoms on the surface. Interestingly, the positions of the Ru atoms followed the periodic arrangement of the Mn sites in crystalline α‐MnO_2_, triggering the reconstruction of small Ru ensembles into large patches of Ru atom arrays. The Ru atom array consisted of symmetric Ru sites that favor the reaction pathway suggested by OPM. Aberration‐corrected high‐angle annular dark‐field scanning transmission electron microscopy (HAADF‐STEM) results showed that the interatomic Ru─Ru distance in Ru/MnO_2_ (2.9 Å) is shorter than that in RuO_2_ (3.1 Å), allowing direct O─O radical coupling (**Figure** **2**a). Consequently, Ru/MnO_2_ exhibited a low overpotential of 161 mV at a current density of 10 mA cm^−2^ and long‐term durability of 200 h (Figure 2b,c). Extensive ex situ and in situ measurements revealed that OER proceeded via the OPM pathway without forming OOH* on Ru/MnO_2_. Theoretical studies confirmed that OER proceeded on Ru/MnO_2_ exhibited a lower energy barrier via the OPM pathway than the conventional AEM pathway (Figure 2d). Similarly, Chang et al. reported periodically arranging Ru atomic arrays on Co_3_O_4_ by substituting surface lattice Co atoms with Ru atoms.^[^ ^40^ ^]^ The Ru atom arrays successfully suppressed the participation of lattice oxygen in OER, preventing the dissolution of active Ru. The resulting Ru array‐Co_3_O_4_ electrocatalyst broke the activity‐stability trade‐off, demonstrating an overpotential of 160 mV at 10 mA cm^−2^ with a stability of 1500 h in 0.5 m H_2_SO_4_. Apart from engineering Ru‐based materials to catalyze OER via OPM pathway, Kumar et al. fabricated short‐range Ir single‐atom‐ensembles (Ir_SAEs_) on Mn‐substituted spinel Co_3_O_4_ (CMO), showing excellent mass activity and significantly improved stability than conventional Ir single atoms supported on Co_3_O_4_ for acidic OER.^[^ ^15^ ^]^ Doping Mn into octahedral sites of Co_3_O_4_ substantially reduced the migration energy barrier for Ir single‐atoms on the CMO surface compared to Co_3_O_4_, facilitating the formation of Ir_SAEs_ during synthesis. The rigid Ir_SAEs_ with appropriate Ir─Ir distance stabilized on the CMO surface not only effectively promoted direct O─O radical coupling but also suppressed lattice oxygen participation, resulting in high OER activity and boosted stability by mitigating Ir‐dissolution.

Apart from the requirement of symmetric dual‐metal sites with appropriate atomic distances, direct O─O coupling can also be realized by weakening intermediate bindings by increasing oxygen coverage on catalyst surfaces. For example, Lin et al. reported an Ir‐Mn‐O*_v_* atomic grid with high‐density Ir sites on reactive MnO_2−_ *_x_* support that mediates oxygen coverage‐enhanced OER via OPM.^[^ ^39^ ^]^ In situ and ex situ characterizations combined with theoretical calculations confirmed that initial coordination defects of Mn─O*_v_* played a key role in the electrochemical generation of enriched oxygen coverage localized within the Ir atomic grids, where highly electrophilic Ir─O^(II−𝛿)−^ bonds proceeded during OER. Therefore, MnO_2−_ *_x_* inside the grids served as an oxygen pool, and O* radicals interacted with electrophilic Ir─O^(II−𝛿)−^ species, facilitating the direct O─O coupling. Accordingly, the resultant Ir‐Mn‐O*_v_* delivered an ultralow overpotential of 166 mV at 10 mA cm^−2^ and maintained a high stability at 100 mA cm^−2^ with a decay rate of only ≈0.3 mV h^−1^ over 180 h tests.

Although engineering the distance of active sites and the coverage of intermediate species can realize the OPM pathway, several challenges remain. The short distance among active sites makes metal atoms prone to aggregate under harsh oxidizing reaction conditions, especially under high current densities and long‐term stability tests. This would change geometric configurations and electronic properties of active sites, possibly switching reaction pathways from those suggested by OPM to others indicated by AEM or LOM. It would be beneficial to better stabilize active sites by enhancing the metal–support interactions, which may be achieved by introducing defects into support materials or using more robust support materials. On the other hand, it is also desirable to develop advanced in situ characterization techniques to monitor the dynamic evolution of active sites, including the changes in their distance during OER, which improves our understanding of reaction pathways and eventually leads to better catalyst designs.

### 3.2 Metal Oxides

Transition metal oxides have been extensively investigated as electrocatalysts for OER due to their cost‐effectiveness and abundance.^[^ ^41^, ^42^ ^]^ In general, constructing interfaces between two or more metal oxide components in heterogeneous structures is an efficient approach to regulate geometries and electronic structures of active sites in metal oxides to drive OER along the reaction pathway suggested by OPM. For example, Zhu et al. developed a Co_3_O_4_/RuCoO_x_ heterojunction structure with an asymmetrical octahedral Ru‐O‐Co coordination environment.^[^ ^19^ ^]^ The strong electron coupling effect between interfacial Ru (RuCoO_x_) and Co (Co_3_O_4_) atoms efficiently catalyzed acidic OER via the OPM pathway, showing an overpotential of 190 mV at a current density of 10 mA cm^−2^. Meanwhile, the electron transfer from surrounding electron‐enriched tetrahedral Co atoms via bridging oxygen bonds helped suppress the overoxidation and subsequent dissolution of active Ru and Co species. This mechanism contributes to the stabilization of Ru‐O‐Co units during OER.

**Figure 3:**
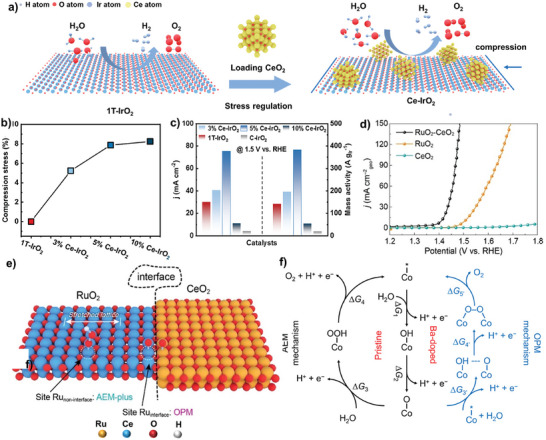
a) Loading CeO_2_ particles on 1T‐IrO_2_ to switch the OER mechanism from AEM to OPM via strain engineering. b) Calculated strain of IrO_2_ using the change rate of Ce‐IrO_2_ relative to 1T‐IrO_2_ crystal spacing. c) Current density and mass activity of Ce‐IrO_2_ and references at 1.5 V versus RHE. Reprinted with permission.^[^ ^18^ ^]^ Copyright 2024, ACS Publishing Group. d) Polarization curves of RuO_2_‐CeO_2_ and references in 0.5 m H_2_SO_4_. e) Schematic of active sites and pathways at the interface and noninterface for RuO_2_‐CeO_2_. Reprinted with permission.^[^ ^46^ ^]^ Copyright 2024, ACS Publishing Group. f) The reaction pathway suggested by AEM on Co_3_O_4_ and OPM on Co_3‐x_Ba_x_O_4_ in acidic electrolyte. Reprinted with permission.^[^ ^17^ ^]^ Copyright 2023, ACS Publishing Group.

The formation of heterostructures often induces synergistic effects (e.g., electronic and strain effects) between metal oxide phases, which may alter OER pathways.^[^ ^43^, ^44^, ^45^ ^]^ For example, Yu et al. fabricated a 3D/2D multiphase catalyst by loading CeO_2_ on metastable 1T phase iridium oxide (Ce‐IrO_2_) with different strains (**Figure** **3**a).^[^ ^18^ ^]^ The optimal catalyst with 8% strain showed an overpotential of 194 mV at 10 mA cm^−2^ and stable operation for 90 h in 0.5 m H_2_SO_4_ (Figure 3b,c). Characterization by Fourier transform infrared (FTIR) spectroscopy, and theoretical simulation results showed that the CeO_2_‐induced strained 1T‐IrO_2_ allowed the occurrence of direct O─O radical coupling via the OPM pathway, different from the traditional AEM pathway on pure 1T‐IrO_2_. Similarly, Song et al. constructed a RuO_2_‐CeO_2_ heterostructure electrocatalyst, showing a high activity with an overpotential of only 180 mV at 10 mA cm^−2^ and long‐term stability for 1000 h in 0.5 m H_2_SO_4_ (Figure 3d).^[^ ^46^ ^]^ Detailed mechanistic investigations revealed that the heterojunction interface and noninterface sites of RuO_2_‐CeO_2_ simultaneously activated reaction pathways suggested by OPM and AEM (Figure 3e). RuO_2_ lattice distortions induced by CeO_2_ promoted the deprotonation of adsorbed *OH atoms near Ru sites, facilitating the stable adsorption of *OOH to lower the energy barrier of the AEM pathway. In addition, Ru and Ce atoms at the interface formed a strong electronic coupling effect through the Ru‐O‐Ce oxygen bridge, allowing direct O─O radical coupling step in OPM. In another approach, doping heteroatoms in metal oxides can alter distances among active sites, resulting in OER along the reaction pathway suggested by OPM.^[^ ^17^, ^47^, ^48^, ^49^ ^]^ For example, Ji et al. fabricated electrocatalysts containing Mn^4−δ^‐O‐Ru^4+δ^ active sites with local structural symmetry and oxidation‐state asymmetric by doping Mn atoms into RuO_2_ host (Mn_0.2_RuO_2_), which successfully switched the reaction pathway from traditional AEM to OPM.^[^ ^49^ ^]^ In situ characterizations and theoretical studies revealed that the charge redistributed Mn^4−δ^‐O‐Ru^4+δ^ units significantly facilitated OH* adsorption, favoring direct coupling of bridge *O─O* intermediate on neighboring Mn/Ru sites. In contrast, rutile RuO_2_ with the Ru‐O‐Ru microstructure catalyzed OER via the AEM pathway. Heteroatom doping and the construction of vacancies in metal oxides may also alter the intrinsic properties of parent metal oxides to activate the OPM pathway.^[^ ^17^, ^50^, ^51^ ^]^ For example, Wang et al. discovered that doping Ba cations into Co_3_O_4_ (Co_3−_ *_x_* Ba*_x_* O_4_) shortened the Co─Co distance and promoted the OH* coverage, facilitating the subsequent deprotonation and then direct O─O coupling step (Figure 3f).^[^ ^17^ ^]^ The resultant Co_3−_ *_x_* Ba*_x_* O_4_ catalyst exhibited an overpotential of 278 mV at 10 mA cm_geo_^−2^ in 0.5 m H_2_SO_4_ with excellent stability over 110 h. In another study, Li et al. reported that confined Gd atoms in MnO_2_ resulted in electron accumulation at Mn─O in asymmetric Gd‐O‐Mn sites with highly active terminal Mn^IV^ = O intermediates and labile O state during OER.^[^ ^52^ ^]^ Theoretical studies indicated that the asymmetric Gd‐O‐Mn units with gradient 4*f*‐2*p*‐3*d* orbital coupling endowed surface unsaturated lattice O sites, which assisted the direct O─O coupling.

These studies indicate that metal oxide catalysts with specific microstructural and electronic structures, such as distance between metal sites, metal valence states, and oxygen states, can drive OER via the reaction pathway suggested by OPM. Notably, Co and Mn oxide‐based electrocatalysts possess various metal oxidation states, enabling them to undergo reversible redox transitions during OER.^[^ ^53^, ^54^, ^55^ ^]^ These transitions are essential for generating reactive oxygen intermediates, such as O*, which subsequently participate in the direct O─O coupling step. In Mn oxide‐based electrocatalysts, the Jahn–Teller distortion may further alter local electronic structures of Mn and induce flexible Mn─O bond strength and angles, allowing tunable distance of active centers for the direct O─O coupling step.^[^ ^56^ ^]^ Furthermore, Co and Mn oxide‐based electrocatalysts exhibit relatively moderate binding energies with O intermediates, having the potential to deliver a theoretical activity comparable to those of RuO_2_ and IrO_2_.^[^ ^57^ ^]^

### 3.3 Metal Oxyhydroxides

**Figure 4:**
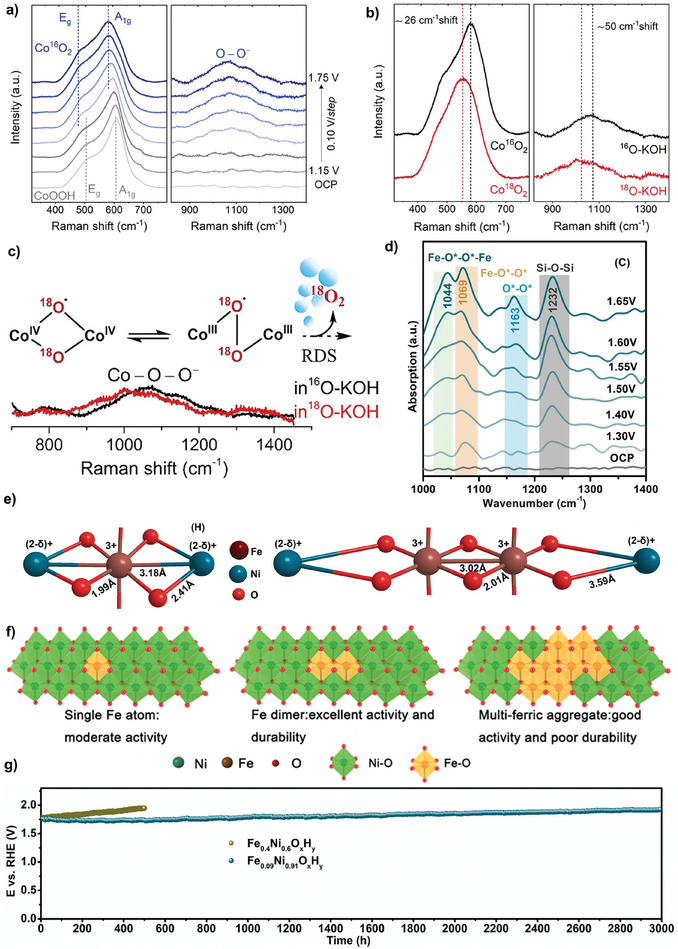
a) In situ Raman spectra of CoOOH at increasing applied potential from open circuit potential to 1.75 V in 0.1 m Fe‐free ^16^O‐KOH. (Left: Co─O bands; right: active oxygen species band). b) In situ Raman spectra of Co^16^O_2_ in 0.1 m Fe‐free ^16^O‐KOH (black) and of Co^18^O_2_ in 0.1 m Fe‐free ^18^O‐KOH (red) recorded at 1.75 V versus RHE. c) Illustration of the O─O coupling step on adjacent Co^4+^O centers. Reprinted with permission.^[^ ^65^ ^]^ Copyright 2020, ACS Publishing Group. d) In situ attenuated total reflectance‐FTIR spectra from 1000 to 1400 cm^−1^ with increasing potentials. e) Illustrating the coordination microenvironments of a single Fe atom in Fe_0.02_Ni_0.98_O*_x_* H*_y_* (left) and Fe dimer in Fe_0.09_Ni_0.91_O*_x_* H*_y_* (right) and f) the relationship of OER performances with the configurations of single Fe atom, Fe dimer, and multi‐ferric aggregate in Ni(OH)_2_ layer. g) Chronopotentiometry curves of Fe_0.09_Ni_0.91_O*_x_* H*_y_* and Fe_0.4_Ni_0.6_O*_x_* H*_y_* at 1000 mA cm^−2^ in 1 m KOH electrolyte. Reprinted with permission.^[^ ^73^ ^]^ Copyright 2024, Elsevier Publishing Group.

Earth‐abundant (oxy)hydroxides containing Fe, Co, and Ni have also been extensively studied for OER in alkaline media.^[^ ^58^, ^59^, ^60^ ^]^ Specifically, NiFe (oxy)hydroxides, whose catalytic activity has surpassed those of precious metal oxides, such as IrO_x_ and RuO_x_.^[^ ^61^, ^62^, ^63^, ^64^ ^]^ However, the OER activity of NiFe (oxy)hydroxides is still severely restricted in the AEM. In this regard, Song et al. reported a novel Fe─Ni oxide catalyst, showing an overpotential of 215 mV at a current density of 10 mA cm^−2^ in 1 m KOH electrolyte.^[^ ^48^ ^]^ Operando spectroscopic studies revealed the formation of heterostructures consisting of nanoclusters of *γ*‐FeOOH covalently linked to a *γ*‐NiOOH support. Theoretical studies suggested that Fe atoms in *γ*‐FeOOH acted as the oxygen‐evolving center and a nearby terrace O site in *γ*‐NiOOH supported oxides as a hydrogen acceptor, allowing for direct O─O radical coupling to deliver catalytic activity enhancement. Nevertheless, the assignment of active centers for OPM is difficult due to the existence of both Ni and Fe in NiFe (oxy)hydroxides. To this end, Moysiadou et al. reported spectroscopic and electrokinetic evidence to validate the occurrence of the OPM pathway on Co oxyhydroxide (CoOOH).^[^ ^65^ ^]^ In detail, operando X‐ray absorption spectroscopy (XAS) results revealed that CoOOH underwent surface reconstruction to form CoO_2_ containing Co^4+^ species. In situ Raman spectroscopy and ^18^O labeling experiments revealed the formation of superoxide intermediates (CoOO^−^) under increasing applied potentials, indicating that lattice oxygen and active oxygen species exchanged with the electrolyte during OER (**Figure** **4**a,b). These superoxide species on adjacent Co centers chemically interacted to generate O_2_ molecules (Figure 4c). Similar phenomena have been experimentally and theoretically observed in Co phosphates and Ni oxides.^[^ ^66^, ^67^, ^68^, ^69^ ^]^ This observation underscores the importance of increased oxidization states in promoting direct O─O radical coupling in OPM.

Besides focusing on enhancing the catalytic activity of NiFe (oxy)hydroxides, the metal leaching at high current densities compromised their stability in alkaline OER.^[^ ^70^ ^]^ For example, Fe ions can be easily oxidized to soluble Fe^4+^ and Fe^6+^ species.^[^ ^71^, ^72^ ^]^ Particularly in the LOM pathway, the continuous surface‐ and bulk‐oxygen transfer further accelerated the Fe dissolution. Developing OPM‐based NiFe (oxy)hydroxides is a promising approach to address these challenges and realize alkaline water electrolysis under industrial conditions. For example, Jia et al. reported a series of Fe*_z_* Ni_1−_ *_z_* O*_x_* H*_y_* (*z*: 0–0.4) electrocatalysts with varying Fe configurations via in situ electrochemical delamination.^[^ ^73^ ^]^ The chemical integration of Fe dimers with Ni(OH)_2_ nanosheets resulted in optimal electronic structures and coordination environments in Fe_0.09_Ni_0.91_O*_x_* H*_y_*, thus accelerating deprotonation and oxygen radical coupling via the OPM pathway (Figure 4d–f). Besides, the extended Ni─O shells surrounding Fe dimers created a locally flexible reaction microenvironment that prevented Fe dissolution at dynamic catalyst/electrolyte interfaces, ensuring long‐term stability at industrial level current densities. The resulting Fe_0.09_Ni_0.91_O*_x_* H*_y_* demonstrated high catalytic activity with an overpotential of 337.1 mV at an industrial level current density of 1000 mA cm^−2^ and robust stability of 125 days in 1 m KOH electrolyte (Figure 4g). These findings provide valuable insights into the structural design and tactical delamination of NiFe (oxy)hydroxide electrocatalysts for OER.

The studies of metal oxyhydroxide catalysts underscore the value of tailoring electronic structures and coordination environments to promote the OPM pathways and mitigate catalyst degradation. Integrating heterostructures with locally adaptable reaction microenvironments provides a potential solution for designing OPM‐based electrocatalysts with high activity and robust durability.

### 3.4 Perovskite Oxides

Perovskite oxides have ABO_3_ structures, where “A” is typically cations of alkaline‐earth metals (e.g., Ba or Sr) or rare‐earth metals (e.g., Pr or La). The “B” is usually transition metals, such as Co, Cu, Ir, or Ru, which forms sixfold octahedra BO6 with oxygen atoms.^[^ ^10^ ^]^ Perovskite oxides are potential OER catalysts owing to their tunable bond strength between their catalytically active sites and reaction intermediates. However, regular perovskite oxides generally catalyze OER via reaction pathways suggested by AEM or LOM.^[^ ^74^, ^75^, ^76^, ^77^, ^78^, ^79^ ^]^

**Figure 5:**
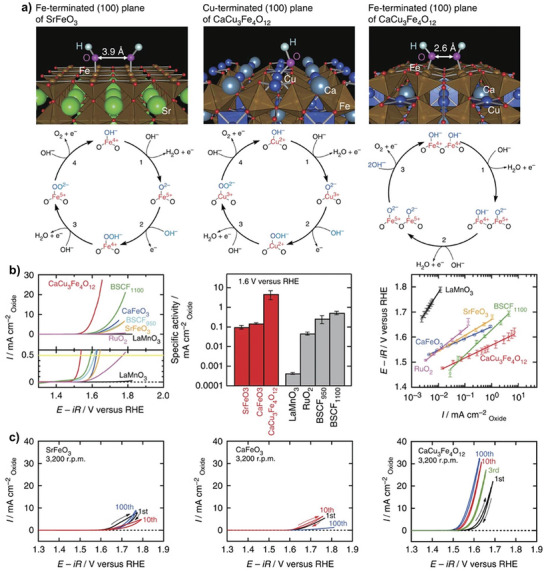
a) OH^−^ adsorbates on Fe and Cu‐terminated (100) planes of SrFeO_3_ and CaCu_3_Fe_4_O_12_, respectively. b) Polarization curves, current density at 1.6 V versus RHE, Tafel plots of OER on CaCu_3_Fe_4_O_12_ and reference catalysts in 0.1 m KOH electrolyte. c) Cyclic voltammograms of OER on CaCu_3_Fe_4_O_12_ and reference catalysts over 100 cycles. Reproduced with permission.^[^ ^14^ ^]^ Copyright 2015, Nature Publishing Group.

Recently, Yagi et al. reported that CaCu_3_Fe_4_O_12_ catalyzed OER via the OPM pathway.^[^ ^14^ ^]^ After incorporating Fe^4+^ and Cu^2+^ into CaCu_3_Fe_4_O_12_, the Fe─O─Fe bonds were heavily bent, resulting in a shortened O─O (connected to the nearest neighboring Fe ions) distance of ≈2.6 Å, which then enabled the occurrence of the direct O─O coupling (**Figure** **5**). In contrast, the O─O distance in SrFeO_3_ was ≈3.9 Å, making it hard to form oxygen molecules. OER on CaCu_3_Fe_4_O_12_ involved only O* and OH* species as intermediates without generating OOH*. The deprotonation of oxyhydroxide groups to form peroxide ions was bypassed, resulting in high OER activity after breaking the original scaling relationships among OER intermediates. Similarly, Yamada et al. found that a structural transformation from simple LaMnO_3_ to quadruple LaMn_7_O_12_ perovskite drastically increases OER catalytic activity.^[^ ^80^ ^]^ The specific activity for LaMn_7_O_12_ was about 30 times larger than that of LaMnO_3_ at 1.7 V in 0.1 m KOH electrolyte. The improved activity was ascribed to the occurrence of the OPM pathway, where the neighboring connected to A′‐ and B‐site Mn ions effectively promoted the direct O─O coupling.

The development of OPM‐based perovskite catalysts is still in fancy. Notably, the choice of doping elements into perovskite oxides plays a critical role in activating the OPM pathway. More studies would be needed in this area. Furthermore, perovskite oxides usually undergo severe surface reconstruction during OER. Understanding their dynamic surface evolution would be the key to designing efficient OPM‐based perovskite electrocatalysts for OER.

### 3.5 Molecular Complexes

**Figure 6:**
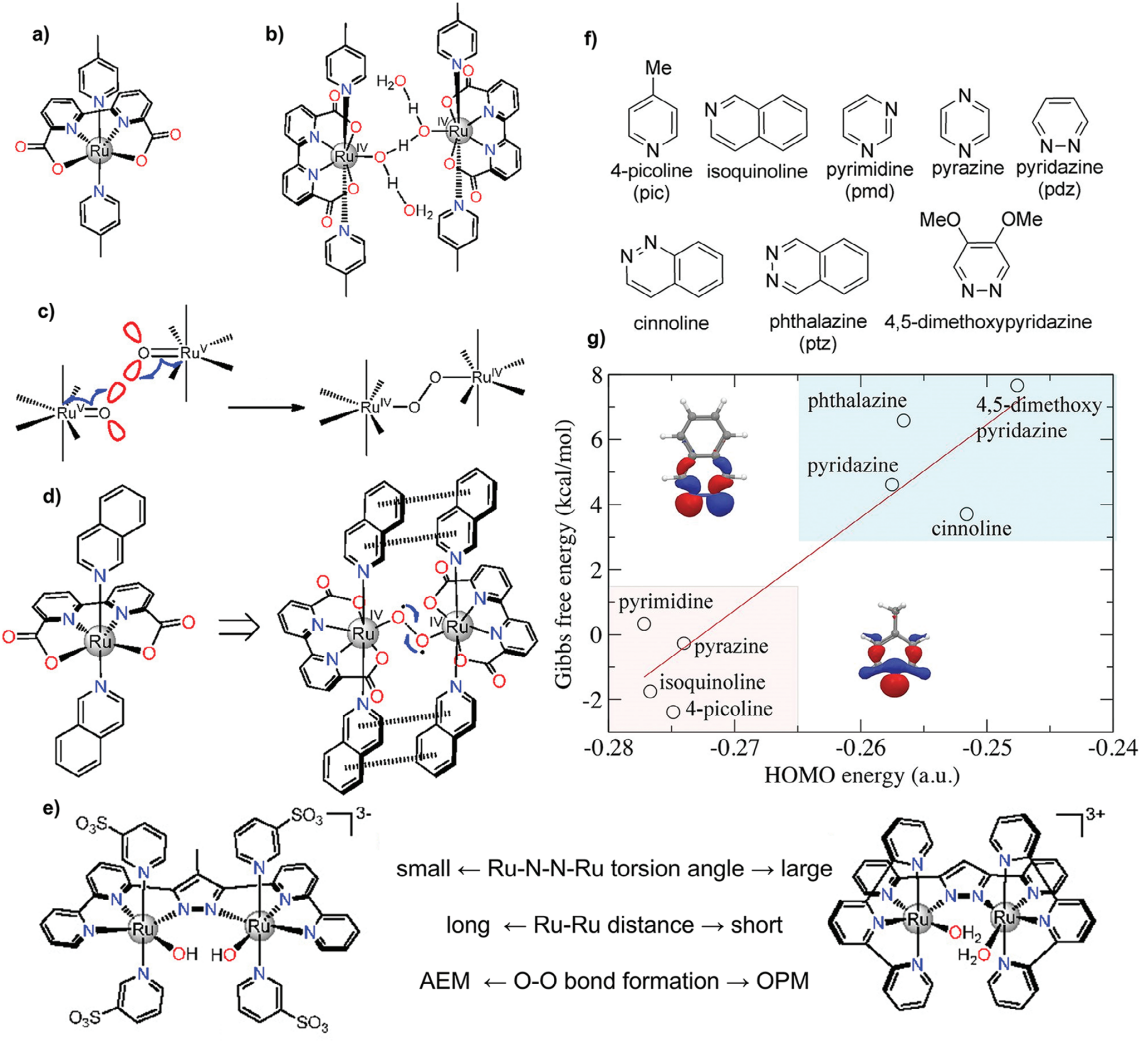
a) Molecular structure of Ru(bda)(pic)_2_. b) Isolated seven‐coordinated Ru(IV) dimer complex with [H_2_O‐HO‐H‐OH‐H_2_O]^−^ bridge. c) Intramolecular coupling between two metal oxo/oxyl units (I2 M) O─O bond formation mechanism between two seven‐coordinated RuV = O (or RuIV‐O•) intermediates. Reproduced with permission.^[^ ^87^ ^]^ Copyright 2012, ACS Publishing Group. d) Molecular structure of 22, and π–π stacking noncovalent interaction between two isoquinoline moieties. Reproduced with permission.^[^ ^89^ ^]^ Copyright 2012, Nature Publishing Group. e) Schematic diagram of different ligands that regulate the OER pathway. Reproduced with permission.^[^ ^90^ ^]^ Copyright 2014, ACS Publishing Group. f) Theoretically screened ligands. g) Gibbs free energy of reaction in pH 0 aqueous solution at 298 K as a function of HOMO energy of ligand in vacuum (in the inset are the HOMO orbital of 4‐picoline and the HOMO‐1 orbital of phthalazine calculated with DFT). Reproduced with permission.^[^ ^91^ ^]^ Copyright 2012, Nature Publishing Group.

Homogeneous catalysts, such as various metal complexes, have been extensively studied as catalysts for OER.^[^ ^42^, ^81^, ^82^, ^83^, ^84^, ^85^ ^]^ Their reaction mechanism can be studied at molecular levels with well‐established spectroscopic techniques. A recent study reported by Masaoka et al. showed that a pentanuclear iron complex efficiently catalyzed OER with an ultrahigh turnover frequency of 1900 s^−1^.^[^ ^86^ ^]^ Theoretical calculations suggested that the presence of adjacent active sites facilitated direct O─O bond coupling with a low reaction energy barrier. Several studies reported that ligands in molecular complexes played a significant role in regulating the reaction pathway of OER. For example, Duan et al. reported a mononuclear Ru complex [Ru(bda)(pic)_2_], consisting of a tetradentate ligand with anionic carboxylate groups (**Figure** **6**a).^[^ ^87^ ^]^ The seven‐coordinated Ru^IV^ dimeric intermediate complex was bridged by a [H_2_O‐HO‐H‐OH‐H_2_O]⁻ moiety, which was proposed to serve as a proton‐conducting channel during OER (Figure 6b). Spectroscopic results showed the oxidation process involving Ru^II^‐OH_2_ → Ru^III^‐OH_2_ → Ru^IV^‐OH → Ru^V^ = O. The close proximity of the two Ru(V) = O species effectively facilitated the direct O─O coupling (Figure 6c). In contrast, the mononuclear six‐coordinated Ru complex‐mediated O─O bond formation generally favors the traditional AEM pathway.^[^ ^88^ ^]^ Interestingly, the rate of direct O─O coupling could be further increased by introducing axial isoquinoline (isoq) ligands into the Ru complex (denoted as [Ru(bda)(isoq)_2_]) (Figure 6d).^[^ ^89^ ^]^ A similar phenomenon was also observed in a binuclear Ru complex with different ligands (Figure 6e).^[^ ^90^ ^]^

Notably, molecular complex catalysts usually suffer from rapid deactivation during OER. Duan et al. identified the dissociation of axial ligands as the primary factor contributing to the instability of mononuclear Ru complexes.^[^ ^91^ ^]^ They proposed that axial ligands with a high energy in their highest occupied molecular orbit could enhance the stability of Ru complexes in OER (Figure 6f,g).

So far, the reported molecular complex OER catalysts show lower catalytic activity than metal oxide and metal oxyhydroxide catalysts, which generally cannot perform well in water‐rich solutions, severely limiting their practical applications. Research strategies, such as engineering ligand configurations, tuning metal centers, and integrating molecular catalysts with conductive supports, should be developed to improve the activity and stability of molecular complex OER catalysts, paving the way for their water oxidation and other catalytic applications.

**Table** **1** summarizes the OER catalytic performances of recently reported catalysts for OER that proceed via the OPM pathway. Although considerable progress has been made in achieving both active and stable operations, attaining industrial‐level performance remains a considerable challenge. Consequently, further innovations are essential to overcome these obstacles and close the gap between laboratory‐scale successes and industrial requirements. Critical issues include enhancing the intrinsic catalytic activity, ensuring long‐term stability under rigorous industrial conditions, and minimizing the overpotential needed to achieve high current densities. Furthermore, challenges related to scalability and cost efficiency must be addressed to facilitate the practical implementation of these catalysts.

**Table 1:** Summary of reported OPM‐based OER catalysts and the corresponding catalytic performance.

Category	Design strategy	Catalyst	Tafel slope [mV dec^−1^]	Overpotential [mV]	Stability [h]	Refs.
Atomic ensembles	Shorten active center distances	Ru/MnO_2_	29.4	161@10 mA cm^−2^	200@10 mA cm^−2^	[13]
	Shorten active center distances	Ir_SAEs_/ CMO	90	235@10 mA cm^−2^	25@10 mA cm^−2^	[15]
	Shorten active center distances	Ru array‐Co_3_O_4_	46	160@10 mA cm^−2^	1500@10 mA cm^−2^	[16]
	Increase intermediates coverage	Ir‐Mn‐O_v_	36	166@10 mA cm^−2^	180@100 mA cm^−2^	[39]
Metal oxides	Heterojunction interface	Co_3_O_4_/RuCoO_x_	77.6	190@10 mA cm^−2^	36@10 mA cm^−2^	[19]
	Heterojunction interface	RuO_2_‐CeO_2_	58.9	180@10 mA cm^−2^	1000@10 mA cm^−2^	[46]
	Asymmetric active sites engineering	P‐Gd SAs@MnO_2_	161.9	281@10 mA cm^−2^	25@10 mA cm^−2^	[52]
	Asymmetric active sites engineering	Mn_0.2_RuO_2_	57	188@10 mA cm^−2^	150@10 mA cm^−2^	[49]
	Shorten active center distances	Co_3‐x_Ba_x_O_4_	41	278@10 mA cm^−2^	110@10 mA cm^−2^	[17]
	Strain engineering	Ce‐IrO_2_	–	194@10 mA cm^−2^	90@10 mA cm^−2^	[18]
Metal oxyhydroxides	Heterostructure	AC‐FD‐NiO*_x_*‐Fe	–	215@10 mA cm^−2^	36@10 mA cm^−2^	[48]
	Asymmetric active site engineering	Fe_0.09_Ni_0.91_O_x_H_y_	41	298@500 mA cm^−2^	3000@1000 mA cm^−2^	[73]
	Asymmetric active site engineering	CFMO‐2	54.4	217@500 mA cm^−2^	1000@10 mA cm^−2^	[92]
Perovskites	Shorten active center distances	CaCu_3_Fe_4_O_12_	44	–	100 cycles	[14]
	Shorten active center distances	LaMn_7_O_12_	100	300@0.01 mA cm^−2^	–	[80]

## 4 *Operando*/In Situ Characterization Techniques

*Operando* and in situ characterization techniques are essential to monitor the variation of structural and electronic properties of catalytic active sites in OER electrocatalysts and the evolution of reaction intermediates during OER. These characterization results provide insights into the OER mechanism on different catalysts and are key to designing high‐performance OPM‐based catalysts. Several commonly used characterization techniques and their recent findings on electrocatalysts catalyzing OER along the reaction pathway suggested by OPM are highlighted in the following subsections.

### 4.1 X‐Ray Absorption Spectroscopy

**Figure 7:**
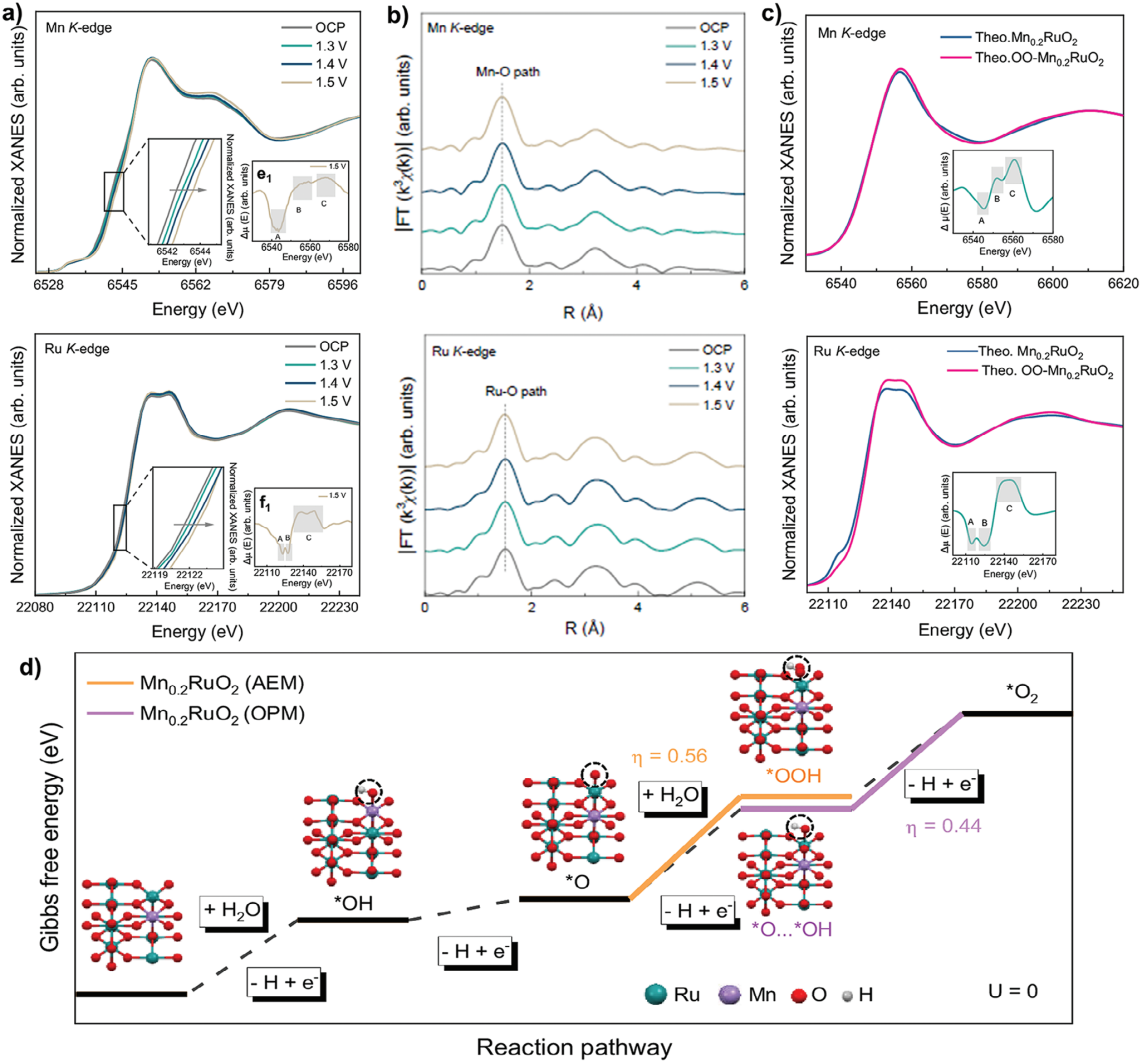
a) *Operando* Mn and Ru K‐edge XANES spectra (Inset: magnified XANES and ΔXANES spectra) and b) the corresponding FT‐EXAFS spectra under applied potentials. c) Theoretical Mn and Ru K‐edge spectra of Mn_0.2_RuO_2_ and OO‐Mn_0.2_RuO_2_ (with adsorbed *O─O* intermediates). d) Gibbs free energy diagram for AEM and OPM pathway on Mn_0.2_RuO_2_. Reprinted with permission.^[^ ^49^ ^]^ Copyright 2024, Nature Publishing Group.

*Operando* XAS can capture the changes in the local environment and electronic structures of active sites during reactions. Specifically, X‐ray absorption near edge structure (XANES) reveals variations in oxidation states and electronic structures of active sites.^[^ ^58^, ^93^, ^94^, ^95^ ^]^ Meanwhile, extended X‐ray absorption fine structure (EXAFS) provides information about the local coordination geometry and bond distance changes.^[^ ^96^ ^]^ For example, Ji et al. utilized *operando* XAS to uncover active sites and reaction pathways on Mn_0.2_RuO_2_ during OER.^[^ ^49^ ^]^ Ru and Mn K‐edge XANES spectra showed a gradual positive shift in the absorption edge with increasing applied potential, indicating increased Ru and Mn oxidation states, attributing to partial electrons transfer from Ru/Mn atoms to absorbed oxygen‐containing species (**Figure** **7**a). Interestingly, the Ru─O and Mn─O bond lengths remained nearly unchanged with increased applied potentials, as indicated by Ru and Mn K‐edge FT‐EXAFS spectra, implying that lattice oxygen hardly participates in OER (Figure 7b). The Mn and Ru K‐edge XANES spectra were further simulated by examining the adsorption of different oxygen‐containing intermediates (e.g., *O, OOH*). The results showed that both Mn and Ru atoms were involved in the adsorption of oxygen‐containing species and formed a characteristic Mn‐*O‐O*‐Ru configuration of Mn_0.2_RuO_2_, indicating the occurrence of OPM pathway during OER (Figure 7c,d). Likewise, combining *operando* XAS and theoretical analysis, Kumar et al. also reported that the short Ir─Ir interatomic distance in Ir_SAE_‐CMO facilitated direct O*─O* radical coupling via the OPM pathway with a lower energy barrier than the AEM pathway.^[^ ^15^ ^]^

The *operando* XAS has been used universally across various materials and is becoming increasingly indispensable in understanding the structure–performance relationship in catalysts.^[^ ^97^, ^98^, ^99^ ^]^ However, XAS is a bulk‐sensitive technique that provides average properties.^[^ ^100^ ^]^ This makes it challenging to uncover the subtle surface structure changes where reactions occur, potentially resulting in a less reliable understanding of reaction mechanisms. To address this limitation, complementary techniques such as ambient pressure X‐ray photoelectron spectroscopy, which is surface‐sensitive, can be used alongside XAS to directly probe the dynamic evolution of active species on catalyst surfaces.^[^ ^101^ ^]^ Meanwhile, emerging data analysis tools, such as machine learning algorithms, may deconvolute XAS signals, isolating surface‐specific changes from bulk material property changes.

### 4.2 Fourier Transform Infrared Spectroscopy

*Operando* FTIR spectroscopy is widely used to study the catalyst surface interfacial properties based on the interaction of electromagnetic radiation and reaction intermediates with permanent or induced dipole moments.^[^ ^102^ ^]^ This technique is sensitive to molecular vibrations of reaction intermediates. It can discriminate geometrical distortions in adsorbed molecules, providing nearly real‐time visualization of reaction intermediates and solution species under working conditions.^[^ ^103^, ^104^ ^]^ These unique features make it valuable to study OER.^[^ ^18^, ^49^, ^105^ ^]^

**Figure 8:**
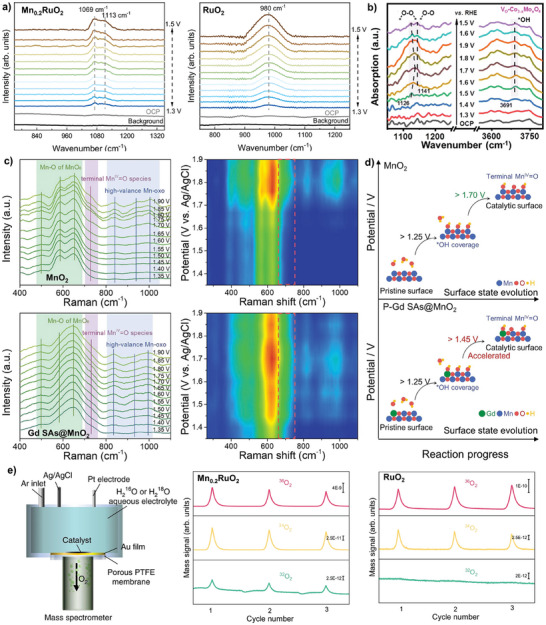
a) *Operando* SR‐FTIR spectra of Mn_0.2_RuO_2_ and p‐RuO_2_ at various applied potentials versus RHE in 0.5 m H_2_SO_4_. Reprinted with permission.^[^ ^49^ ^]^ Copyright 2024, Nature Publishing Group. b) In situ FTIR spectra of V_O_‐Mo*_x_* Co_3−_ *_x_* O_4_ at various applied potentials versus RHE in 0.1 m HClO_4_ electrolyte. Reprinted with permission.^[^ ^47^ ^]^ Copyright 2024, Royal Society of Chemistry. c) In situ Raman spectra and the corresponding contour plot of MnO_2_ and Gd SAs@MnO_2_. d) Schematic comparison between the surface evolution of MnO_2_ and Gd SAs@MnO_2_. Reprinted with permission.^[^ ^52^ ^]^ Copyright 2024, Elsevier. e) *Operando* DEMS Mn_0.2_RuO_2_ and p‐RuO_2_. Reprinted with permission.^[^ ^49^ ^]^ Copyright 2024, Nature Publishing Group.

Utilizing *operando* synchrotron radiation FTIR (SR‐FTIR), Ji et al. probed the OER mechanism on Mn_0.2_RuO_2_.^[^ ^49^ ^]^ Distinctive vibration frequencies at 1113 cm^−1^ emerged as the applied potential gradually increased from 1.3 to 1.5 V, which was attributed to the bridging oxygen configuration in Mn‐O‐Ru units. The potential‐dependent SR‐FTIR absorption band indicated the formation of *O─O* intermediates with a characteristic M‐*O‐O*‐M configuration, suggesting that OER proceeded on Mn_0.2_RuO_2_ via the OPM pathway (**Figure** **8**a). A single broad infrared vibration band at 980 cm^−1^ emerged when the applied potential increased from 1.3 to 1.5 V on RuO_2_, which was attributed to a typical Ru‐*OOH intermediate in the traditional AEM pathway. Similarly, Sun et al. applied *operando* electrochemical FTIR spectroscopy to investigate the OER mechanism on V_O_‐Mo*_x_* Co_3−_ *_x_* O_4_. Two distinctive adsorption bands at 1126 and 1141 cm^−1^ were detected at an applied potential of >1.5 V versus RHE (Figure 8b). The vibration band at 1126 cm^−1^ was assigned to the bridged oxygen species (*─O─O─*) between neighboring metal sites, and the peak at 1141 cm^−1^ was related to the linearly bound superoxide species (*─O─O) before releasing an O_2_ molecule. These FTIR results supported the occurrence of the OPM pathway on V_O_‐Mo*_x_* Co_3−_ *_x_* O_4_ during OER in acidic electrolytes. In contrast, these characteristic peaks were not detected on Co_3_O_4_, indicating a typical AEM pathway.

Although *operando* FTIR can provide direct information about reaction intermediates and solution species under working conditions, there are multiple challenges. First, the accurate assignment of oxygenated species is still debatable. For example, there is a great discrepancy in assigning the vibration band of O─O intermediates (900,^[^ ^19^ ^]^ 1069,^[^ ^49^ ^]^ and 1087 cm^−1^,^[^ ^18^ ^]^ in different studies). Second, directly comparing characteristic vibration bands from different electrocatalysts may be questionable because of the difference in intrinsic structure of electrocatalysts, their structure evolution during OER, and the external testing environment (e.g., electrolyte and reaction‐inducing factors). Third, severe gas bubble formation at higher potentials in OER may also affect the accuracy of collected spectra.

### 4.3 Raman Spectroscopy

Raman spectroscopy can be a surface‐sensitive technique for detecting active species on catalyst surfaces, and it has been used to uncover OER mechanisms.^[^ ^42^, ^106^, ^107^ ^]^ In particular, in situ Raman can be used to characterize vibration modes of oxygen species, such as O─O bonds, lattice oxygen, and adsorbed intermediates.^[^ ^108^, ^109^, ^110^, ^111^, ^112^ ^]^ It may also be used to track the structural evolution of electrocatalysts during OER.^[^ ^113^, ^114^ ^]^ For example, Li et al. investigated the chemical state and geometric structure variation of local Mn─O units in Gd‐doped MnO_2_ (Gd SAs@MnO_2_).^[^ ^52^ ^]^ Specifically, on regular MnO_2_, the peak between 570 and 590 cm^−1^ was associated with in‐plane Mn─O stretching vibrations along octahedral chain layers (Figure 8c). However, on Gd SAs@MnO_2_, this peak was significantly reduced due to the disruption of the Mn─O chain by forming localized Gd‐O‐Mn. Meanwhile, a potential‐depended signal peak, attributing to the surface terminal Mn^IV^ = O intermediates, appeared at a low bias potential of 1.45 V versus RHE on Gd SAs@MnO_2_. The formation of Gd‐O‐Mn facilitated electron accumulation at the terminal of Mn─O and accelerated the formation of a labile O state. Theoretical studies suggested that the presence of an unsaturated surface lattice O site with labile properties could promote direct O─O coupling via the OPM pathway (Figure 8d).

In situ Raman spectroscopy was also used to identify surface‐adsorbed molecules or intermediates in OER.^[^ ^115^ ^]^ Furthermore, labeling intermediates with isotopes and characterizing them with in situ Raman spectroscopy enables the acquisition of molecule‐level insights into the OER mechanism.^[^ ^116^ ^]^ For example, Lu et al. performed in situ Raman studies to detect reaction intermediates on Sr‐IrO_x_ catalyst during OER in 0.1 m HClO_4_.^[^ ^50^ ^]^ A peak at around 1050 cm^−1^ was observed under the potential of 1.40 V versus RHE, attributing to the dioxygen radical coupling. This provides direct evidence of the OPM pathway on Sr‐IrO_x_ during OER.

Because Raman spectra are often unaffected by water molecules, they have been used to study reaction mechanisms in aqueous solutions.^[^ ^117^, ^118^, ^119^, ^120^, ^121^ ^]^ Nevertheless, a key challenge of applying in situ Raman spectroscopy to study OER is accurately distinguishing vibrational modes of different oxygen species, particularly lattice oxygen and absorbed OOH* intermediates or O_2_ molecules. Their Raman‐sensitive vibration modes are often overlapped. Besides, some OER electrocatalysts, such as complex oxide materials, may generate strong fluorescence emissions under laser irradiation, strongly interfering with Raman signals.

### 4.4 Differential Electrochemical Mass Spectrometry (DEMS)

Studying electrolytes labeled with isotopes (e.g., ¹⁸O‐labeled electrolyte) by DEMS has been used to explore OER reaction mechanisms.^[^ ^122^, ^123^, ^124^ ^]^ This technique allows qualitative analysis of different oxygen isotopes (e.g., ^32^O_2_, ^34^O_2_, and ^36^O_2_) found in reaction intermediates. For example, detecting ^36^O_2_ implies that OER may proceed via OPM or AEM pathways, with ^36^O_2_ produced entirely from ^18^O in water. Conversely, the appearance of ^34^O_2_ and ^32^O_2_ suggests that OER has proceeded via the LOM pathway. In this scenario, ^34^O_2_ may come from the combination of ^16^O in the lattice oxygen of the catalyst and ^18^O in water, while ^32^O_2_ arises solely from ^16^O of catalyst lattice oxygen. For example, Ji et al. performed *operando* DEMS with isotope labeling to investigate OER mechanisms on Mn_0.2_RuO_2_ in ^18^O‐labeled 0.5 m H_2_SO_4_ aqueous electrolytes. The ^36^O_2_ signal intensity was two and three orders of magnitude higher than ^32^O_2_ and ^34^O_2_ on Mn_0.2_RuO_2_, indicating that the oxygen molecules primarily originated from two ^18^O atoms in the isotope‐labeled electrolyte (Figure 8e). Combining DEMS and FTIR results, the author proposed that OER proceeded on Mn_0.2_RuO_2_ via the OPM pathway. This technique was also used to validate the occurrence of the reaction pathway suggested by LOM.^[^ ^125^ ^]^

DEMS technology still has limitations in studying OER. First, DEMS lacks the temporal resolution to capture short‐lived intermediates effectively, making it difficult to track the rapid transformation reactions intermediates in OER. Second, DEMS is a predominantly surface‐sensitive technology and cannot probe bulk oxygen in catalysts. Integrating DEMS with high‐resolution mass spectrometry enhances detection sensitivity and resolution, which may enable the detection of minor reaction intermediate species and the tracking of catalysts’ dynamic changes. Meanwhile, conducting post‐characterization assessments is crucial to determine whether lattice oxygen participates during OER.

## 5 Conclusions and Perspective

Sluggish OER on current electrocatalysts has made it a roadblock in many energy conversion and storage devices. The reaction pathway via direct O─O coupling suggested by OPM offers the possibility of breaking the trade‐off of catalytic activity and stability. This review summarizes recent progress in developing new electrocatalysts to drive OER along the OPM pathway. Several *operando* characterization tools have played essential roles in identifying catalytically active sites and monitoring reaction intermediates to validate this reaction mechanism and elucidate new electrocatalysts’ structure–performance relationships.

Accordingly, the improved reaction kinetics and reduced overpotentials enabled by new OPM‐based electrocatalysts open the possibility of enhancing the overall hydrogen production efficiency by water electrolysis. Similarly, in various metal‐air batteries, where the OER electrocatalysts play a crucial role during the charging cycle, the stability and activity of these new OPM‐based catalysts may lead to higher energy storage efficiency and longer battery lifetime. Nevertheless, advancements in developing OPM‐based OER electrocatalysts to realize their practical applications require an improved understanding of their reaction mechanisms, materials innovation in designing more active and stable electrocatalysts, and advanced characterization techniques. The following subsections discuss these issues.

### 5.1 Reaction Mechanism

Understanding the structure and performance correlations is critical to designing efficient OPM‐based OER electrocatalysts. However, aggregation of metal species and phase transformation of materials under oxidizing OER conditions continuously change catalysts’ geometric configurations and electronic properties, making it difficult to decipher their reaction pathway accurately. One potential solution is to use model catalysts with well‐defined geometric and electronic structures and tunable active sites to gain insights into reaction mechanisms. For example, dual‐atom electrocatalysts with two adjacent homo‐ or hetero‐metal centers may serve as an excellent platform to unveil reaction mechanisms on their unique active sites. Especially studying the evolution of these dual‐atom active sites using *operando* techniques may provide a good understanding of how changes in active sites affect the critical direct O─O coupling step. On the other hand, understanding the degradation of active sites during OER is essential to designing electrocatalysts with good stability. The stability of active sites could be improved by strengthening interactions between metal active sites and support materials through defect engineering or embedding active sites in robust support materials.

### 5.2 Material Innovation

New materials are needed to produce efficient OPM‐based OER electrocatalysts. Several areas are promising and worth further study.

First, one focus is to use dual‐metal ensembles to promote O─O coupling. The results discussed in this review show that dual‐metal ensembles with optimal interatomic distances can induce favorable interactions between oxygen intermediates, leading to efficient O─O coupling step. They can overcome the limitations of isolated single‐atom sites. Further optimization of the spatial arrangement of dual‐metal ensembles has an excellent chance of yielding more efficient OER electrocatalysts.

Second, controllably incorporating defects (e.g., oxygen vacancy) into metal oxide supports can enhance binding energy with oxygen intermediates and the oxidation states of anchored metal species, substantially improving catalytic performance.^[^ ^39^, ^126^, ^127^ ^]^ For example, defective MnO_2−_ *_x_* has oxygen vacancies that can facilitate the formation of electrophilic oxygen species, resulting in a high oxygen coverage, which is essential for enabling O─O coupling.^[^ ^39^ ^]^

Third, weak metal–support interactions often lead to corrosion and deactivation of metal active sites. Forming nanoscale hybrid materials by bonding electrically conductive materials (e.g., carbon materials) with metal oxides as support materials may enhance metal–support interactions by enhancing electron transport and increasing material structural stability. Stronger metal–support interactions may also regulate spin configurations of metal active sites to accelerate electron transfer and realize optimized reaction intermediate adsorption–desorption behaviors.

Fourth, amorphous materials are currently often used as support materials. Engineering crystalline materials as support materials may provide an opportunity to precisely control distances between catalytically active sites with well‐defined coordinated environments. This may lead to a new class of OPM‐based OER electrocatalysts. Also, developing hybrid electrocatalysts capable of leveraging multiple mechanisms under varying conditions could unlock new possibilities for OER with improved performance.

Fifth, since OER is often conducted in acidic electrolytes. Developing acid‐stable materials is essential to prevent the dissolution of active sites. For example, the combination of active oxides (e.g., RuO_2_, IrO_2_, and Co_3_O_4_) with stable oxides (e.g., TiO_2_, MnO_2_, SnO_2_, Ta_2_O_5_, and Nb_2_O_5_) can prevent the dissolution of the active species, ensuring long‐term catalytic stability.^[^ ^128^, ^129^, ^130^, ^131^ ^]^ Besides, novel synthesis approaches such as 3D printing, microwave synthesis, and chemical vapor deposition offer precise control of active sites’ distance, morphology, and composition, which could be utilized to achieve scale‐up synthesis for industrial applications.^[^ ^132^, ^133^, ^134^, ^135^, ^136^ ^]^

### 5.3 New Characterization Techniques

New characterization techniques can assist in several aspects of the development of efficient OPM‐based OER electrocatalysts.

First, although *operando* XAS, FTIR, and Raman techniques have been used to study OER electrocatalysts, various active phases (e.g., facet, size, and morphology) in catalysts and their external environment (e.g., electrolytes and testing conditions) making it challenging to decipher reaction mechanisms. Further studies must carefully design control experiments to reduce interferences from various variables so reaction mechanisms can be determined more precisely.

Second, isotope labeling has shown a strong capacity to monitor active sites and their surrounding environment. For example, tracking ^18^O and ^16^O atoms in catalysts has enabled the study of different reaction pathways.^[^ ^50^, ^137^, ^138^ ^]^ Combining isotope labeling with *operando* characterization techniques can provide a more in‐depth understanding of reaction mechanisms and catalyst behaviors.

Third, current *operando* characterization techniques typically take minutes to hours to obtain results, suggesting that only (quasi)stable states of reaction intermediates can be monitored. However, detected (quasi)stable reaction intermediates might not be actual rate‐determining steps. Reaction intermediates generally have a lifetime of picoseconds. Thus, new time‐resolved operando characterization techniques are desirable for monitoring the dynamic change of active sites in their structures and oxidation states induced by reaction intermediates so reaction pathways can be explained well. These characterization results can be further integrated with theoretical calculations/simulations to elucidate reaction mechanisms and guide the design of OPM‐based OER electrocatalysts.

## Conflict of Interest

The authors declare no conflict of interest.

## References

[1] I. Katsounaros , S. Cherevko , A. R. Zeradjanin , K. J. Mayrhofer , Angew. Chem., Int. Ed. 2014, 53, 102. 10.1002/anie.201306588 24339359

[2] K. Zeng , D. Zhang , Prog. Energy Combust. Sci. 2010, 36, 307. 10.1016/j.pecs.2009.12.003 PMC3820115 24223466

[3] T. Zhao , X. Shen , Y. Wang , R. K. Hocking , Y. Li , C. Rong , K. Dastafkan , Z. Su , C. Zhao , Adv. Funct. Mater. 2021, 31, 2100614.

[4] T. Zhao , S. Wang , C. Jia , C. Rong , Z. Su , K. Dastafkan , Q. Zhang , C. Zhao , Small 2023, 19, 2208076. 10.1002/smll.202208076 36971280

[5] M. S. A. Sher Shah , G. Y. Jang , K. Zhang , J. H. Park , EcoEnergy 2023, 1, 344.

[6] Y. Shen , W. Li , W. Wang , L. Xin , W. Xiao , G. Xu , D. Chen , L. Wang , F. Liu , Z. Wu , Inorg. Chem. Front. 2024, 11, 5508.

[7] J. Song , C. Wei , Z.‐F. Huang , C. Liu , L. Zeng , X. Wang , Z. J. Xu , Chem. Soc. Rev. 2020, 49, 2196. 32133479 10.1039/c9cs00607a

[8] Y. N. Zhou , W. L. Yu , H. J. Liu , R. Y. Fan , G. Q. Han , B. Dong , Y. M. Chai , EcoEnergy 2023, 1, 425.

[9] H. Wang , P. Yang , X. Sun , W. Xiao , X. Wang , M. Tian , G. Xu , Z. Li , Y. Zhang , F. Liu , J. Energy Chem. 2023, 87, 286.

[10] C. Rong , K. Dastafkan , Y. Wang , C. Zhao , Adv. Mater. 2023, 35, 2211884. 10.1002/adma.202211884 37549889

[11] L. Li , P. Wang , Q. Shao , X. Huang , Adv. Mater. 2021, 33, 2004243. 10.1002/adma.202004243 33749035

[12] X. Wang , H. Zhong , S. Xi , W. S. V. Lee , J. Xue , Adv. Mater. 2022, 34, 2107956. 10.1002/adma.202107956 35853837

[13] C. Lin , J.‐L. Li , X. Li , S. Yang , W. Luo , Y. Zhang , S.‐H. Kim , D.‐H. Kim , S. S. Shinde , Y.‐F. Li , Nat. Catal. 2021, 4, 1012.

[14] S. Yagi , I. Yamada , H. Tsukasaki , A. Seno , M. Murakami , H. Fujii , H. Chen , N. Umezawa , H. Abe , N. Nishiyama , Nat. Commun. 2015, 6, 8249. 26354832 10.1038/ncomms9249 PMC4579779

[15] A. Kumar , M. Gil‐Sepulcre , J. Lee , V. Q. Bui , Y. Wang , O. Rüdiger , M. G. Kim , S. DeBeer , H. Tüysüz , Adv. Mater. 2024, 36, 2401648. 10.1002/adma.202401648 39318088

[16] W. Zhu , F. Yao , K. Cheng , M. Zhao , C.‐J. Yang , C.‐L. Dong , Q. Hong , Q. Jiang , Z. Wang , H. Liang , J. Am. Chem. Soc. 2023, 145, 17995. 37550082 10.1021/jacs.3c05556

[17] N. Wang , P. Ou , R. K. Miao , Y. Chang , Z. Wang , S.‐F. Hung , J. Abed , A. Ozden , H.‐Y. Chen , H.‐L. Wu , J. Am. Chem. Soc. 2023, 145, 7829. 37010254 10.1021/jacs.2c12431

[18] H. Yu , Y. Ji , C. Li , W. Zhu , Y. Wang , Z. Hu , J. Zhou , C.‐W. Pao , W.‐H. Huang , Y. Li , J. Am. Chem. Soc. 2024, 146, 20251. 38996085 10.1021/jacs.4c05204

[19] J. Liang , X. Gao , K. Xu , J. Lu , D. Liu , Z. Zhao , E. C. Tse , Z. Peng , W. Zhang , J. Liu , Small 2023, 19, 2304889. 10.1002/smll.202304889 37438574

[20] M. T. Koper , J. Electroanal. Chem. 2011, 660, 254.

[21] J. H. Montoya , L. C. Seitz , P. Chakthranont , A. Vojvodic , T. F. Jaramillo , J. K. Nørskov , Nat. Mater. 2017, 16, 70. 10.1038/nmat4778 27994241

[22] J. Shan , Y. Zheng , B. Shi , K. Davey , S.‐Z. Qiao , ACS Energy Lett. 2019, 4, 2719.

[23] N. Hodnik , P. Jovanovič , A. Pavlišič , B. Jozinović , M. Zorko , M. Bele , V. S. Šelih , M. Šala , S. Hočevar , M. Gaberšček , J. Phys. Chem. C 2015, 119, 10140.

[24] D. Cao , Z. Zhang , Y. Cui , R. Zhang , L. Zhang , J. Zeng , D. Cheng , Angew. Chem., Int. Ed. 2023, 135, e202214259. 10.1002/anie.202214259 36495017

[25] C. Jia , S. Li , Y. Zhao , R. K. Hocking , W. Ren , X. Chen , Z. Su , W. Yang , Y. Wang , S. Zheng , Adv. Funct. Mater. 2021, 31, 2107072.

[26] H. Tian , A. Song , P. Zhang , K. Sun , J. Wang , B. Sun , Q. Fan , G. Shao , C. Chen , H. Liu , Adv. Mater. 2023, 35, 2210714. 10.1002/adma.202210714 36630970

[27] S. Liu , Q. Meyer , C. Jia , S. Wang , C. Rong , Y. Nie , C. Zhao , Energy Environ. Sci. 2023, 16, 3792.

[28] H. Zhao , R. Yu , S. Ma , K. Xu , Y. Chen , K. Jiang , Y. Fang , C. Zhu , X. Liu , Y. Tang , Nat. Catal. 2022, 5, 818.

[29] J. Sui , H. Liu , S. Hu , K. Sun , G. Wan , H. Zhou , X. Zheng , H. L. Jiang , Adv. Mater. 2022, 34, 2109203. 10.1002/adma.202109203 34883530

[30] X. Chen , A. Xu , D. Wei , F. Huang , J. Ma , H. He , J. Xu , Chin. Chem. Lett. 2025, 36, 110175.

[31] F. Huang , X. Chen , H. Sun , Q. Zeng , J. Ma , D. Wei , J. Zhu , Z. Chen , T. Liang , X. Yin , Angew. Chem. Int. Ed. 2024, 10.1002/anie.202415642.

[32] C. Rong , K. Flint , C. Doonan , Y. Chen , Next Mater. 2025, 7, 100457.

[33] C. Jia , Q. Sun , R. Liu , G. Mao , T. Maschmeyer , J. J. Gooding , T. Zhang , L. Dai , C. Zhao , Adv. Mater. 2024, 36, 2404659. 10.1002/adma.202404659 38870958

[34] C. Rong , X. Shen , Y. Wang , L. Thomsen , T. Zhao , Y. Li , X. Lu , R. Amal , C. Zhao , Adv. Mater. 2022, 34, 2110103. 10.1002/adma.202110103 35384087

[35] J. Shan , C. Ye , S. Chen , T. Sun , Y. Jiao , L. Liu , C. Zhu , L. Song , Y. Han , M. Jaroniec , J. Am. Chem. Soc. 2021, 143, 5201. 33764061 10.1021/jacs.1c01525

[36] M. Zlatar , D. Nater , D. Escalera‐López , R. M. Joy , P. Pobedinskas , K. Haenen , C. Copéret , S. Cherevko , Electrochim. Acta 2023, 444, 141982.

[37] W. Wang , C. Li , C. Zhou , X. Xiao , F. Li , N. Y. Huang , L. Li , M. Gu , Q. Xu , Angew. Chem. Int. Ed. 2024, 63, e202406947. 10.1002/anie.202406947 38650436

[38] Y. Hao , S.‐F. Hung , W.‐J. Zeng , Y. Wang , C. Zhang , C.‐H. Kuo , L. Wang , S. Zhao , Y. Zhang , H.‐Y. Chen , J. Am. Chem. Soc. 2023, 145, 23659. 37871168 10.1021/jacs.3c07777

[39] H. Y. Lin , Q. Q. Yang , M. Y. Lin , H. G. Xu , X. Tang , H. Q. Fu , H. Wu , M. Zhu , L. Zhou , H. Y. Yuan , Adv. Mater. 2024, 36, 2408045. 10.1002/adma.202408045 39177118

[40] J. Chang , Y. Shi , H. Wu , J. Yu , W. Jing , S. Wang , G. I. Waterhouse , Z. Tang , S. Lu , J. Am. Chem. Soc. 2024, 146, 12958. 38695595 10.1021/jacs.3c13248

[41] K. Dastafkan , S. Wang , C. Rong , Q. Meyer , Y. Li , Q. Zhang , C. Zhao , Adv. Funct. Mater. 2022, 32, 2107342.

[42] Y. Xiao , K. Dastafkan , Z. Su , C. Rong , C. Zhao , J. Mater. Chem. A 2023, 11, 19418.

[43] Y. Qin , T. Yu , S. Deng , X.‐Y. Zhou , D. Lin , Q. Zhang , Z. Jin , D. Zhang , Y.‐B. He , H.‐J. Qiu , Nat. Commun. 2022, 13, 3784. 35778401 10.1038/s41467-022-31468-0 PMC9249734

[44] H. Su , C. Yang , M. Liu , X. Zhang , W. Zhou , Y. Zhang , K. Zheng , S. Lian , Q. Liu , Nat. Commun. 2024, 15, 95. 38167374 10.1038/s41467-023-44483-6 PMC10762142

[45] S. Maiti , K. Maiti , M. T. Curnan , K. Kim , K.‐J. Noh , J. W. Han , Energy Environ. Sci. 2021, 14, 3717.

[46] H. Song , X. Yong , G. I. Waterhouse , J. Yu , H. Wang , J. Cai , Z. Tang , B. Yang , J. Chang , S. Lu , ACS Catal. 2024, 14, 3298.

[47] L. Sun , M. Feng , Y. Peng , X. Zhao , Y. Shao , X. Yue , S. Huang , J. Mater. Chem. A 2024, 12, 8796.

[48] F. Song , M. M. Busch , B. Lassalle‐Kaiser , C.‐S. Hsu , E. Petkucheva , M. Bensimon , H. M. Chen , C. Corminboeuf , X. Hu , ACS Cent. Sci. 2019, 5, 558. 30937383 10.1021/acscentsci.9b00053 PMC6439451

[49] Q. Ji , B. Tang , X. Zhang , C. Wang , H. Tan , J. Zhao , R. Liu , M. Sun , H. Liu , C. Jiang , Nat. Commun. 2024, 15, 8089. 39284800 10.1038/s41467-024-52471-7 PMC11405856

[50] Z. Lu , W. Zhang , C. Zhu , Y. Wen , M. Wang , Y. Wang , Angew. Chem. Int. Ed. 2024, 10.1002/anie.202418456.

[51] M. Cui , R. Guo , Y. Zhou , W. Zhao , Y. Liu , W. Luo , Q. Ou , S. Zhang , ACS Catal. 2024, 14, 16353.

[52] M. Li , X. Wang , D. Zhang , Y. Huang , Y. Shen , F. Pan , J. Lin , W. Yan , D. Sun , K. Huang , Nano Energy 2024, 128, 109868.

[53] S. Park , Y. H. Lee , S. Choi , H. Seo , M. Y. Lee , M. Balamurugan , K. T. Nam , Energy Environ. Sci. 2020, 13, 2310.

[54] B. Zhang , L. Sun , Dalton Trans. 2018, 47, 14381. 30129959 10.1039/c8dt01931b

[55] Y. Zhou , S. Sun , C. Wei , Y. Sun , P. Xi , Z. Feng , Z. J. Xu , Adv. Mater. 2019, 31, 1902509. 10.1002/adma.201902509 31361056

[56] K. Jin , H. Seo , T. Hayashi , M. Balamurugan , D. Jeong , Y. K. Go , J. S. Hong , K. H. Cho , H. Kakizaki , N. Bonnet‐Mercier , J. Am. Chem. Soc. 2017, 139, 2277. 28029792 10.1021/jacs.6b10657

[57] S. Trasatti , Electrochim. Acta 1984, 29, 1503.

[58] X. Bo , R. K. Hocking , S. Zhou , Y. Li , X. Chen , J. Zhuang , Y. Du , C. Zhao , Energy Environ. Sci. 2020, 13, 4225.

[59] M. S. Burke , L. J. Enman , A. S. Batchellor , S. Zou , S. W. Boettcher , Chem. Mater. 2015, 27, 7549.

[60] M. B. Stevens , C. D. Trang , L. J. Enman , J. Deng , S. W. Boettcher , J. Am. Chem. Soc. 2017, 139, 11361. 28789520 10.1021/jacs.7b07117

[61] Y. Hao , Y. Li , J. Wu , L. Meng , J. Wang , C. Jia , T. Liu , X. Yang , Z.‐P. Liu , M. Gong , J. Am. Chem. Soc. 2021, 143, 1493. 33439638 10.1021/jacs.0c11307

[62] F. Dionigi , P. Strasser , Adv. Energy Mater. 2016, 6, 1600621.

[63] Z. He , J. Zhang , Z. Gong , H. Lei , D. Zhou , N. Zhang , W. Mai , S. Zhao , Y. Chen , Nat. Commun. 2022, 13, 2191. 35449165 10.1038/s41467-022-29875-4 PMC9023528

[64] S. Wang , K. Dastafkan , S. Wu , Q. Sun , C. Rong , D. Yao , C. Zhao , ACS Catal. 2024, 15, 44.

[65] A. Moysiadou , S. Lee , C.‐S. Hsu , H. M. Chen , X. Hu , J. Am. Chem. Soc. 2020, 142, 11901. 32539368 10.1021/jacs.0c04867

[66] R. R. Rao , S. Corby , A. Bucci , M. García‐Tecedor , C. A. Mesa , J. Rossmeisl , S. Giménez , J. Lloret‐Fillol , I. E. Stephens , J. R. Durrant , J. Am. Chem. Soc. 2022, 144, 7622. 35442661 10.1021/jacs.1c08152 PMC9073940

[67] C. Costentin , D. G. Nocera , Proc. Natl. Acad. Sci. USA 2017, 114, 13380. 28874551 10.1073/pnas.1711836114 PMC5754779

[68] Y. Surendranath , M. W. Kanan , D. G. Nocera , J. Am. Chem. Soc. 2010, 132, 16501. 20977209 10.1021/ja106102b

[69] L.‐P. Wang , T. Van Voorhis , J. Phys. Chem. Lett. 2011, 2, 2200.

[70] G. Liu , Y. Xu , T. Yang , L. Jiang , Nano Mater. Sci. 2023, 5, 101.

[71] C. Feng , X. She , Y. Xiao , Y. Li , Angew. Chem., Int. Ed. 2023, 62, e202218738. 10.1002/anie.202218738 36583473

[72] B. M. Hunter , N. B. Thompson , A. M. Müller , G. R. Rossman , M. G. Hill , J. R. Winkler , H. B. Gray , Joule 2018, 2, 747.

[73] H. Jia , H. Wang , F. Yan , Z. Li , R. Li , S. Li , J. Wang , H. Zhang , Appl. Catal. B 2024, 364, 124861.

[74] X. Liang , L. Shi , Y. Liu , H. Chen , R. Si , W. Yan , Q. Zhang , G. D. Li , L. Yang , X. Zou , Angew. Chem., Int. Ed. 2019, 58, 7631. 10.1002/anie.201900796 30775830

[75] M. Lu , Y. Zheng , Y. Hu , B. Huang , D. Ji , M. Sun , J. Li , Y. Peng , R. Si , P. Xi , Sci. Adv. 2022, 8, eabq3563. 35905191 10.1126/sciadv.abq3563 PMC9337758

[76] J.‐W. Zhao , H. Zhang , C.‐F. Li , X. Zhou , J.‐Q. Wu , F. Zeng , J. Zhang , G.‐R. Li , Energy Environ. Sci. 2022, 15, 3912.

[77] L. Yang , G. Yu , X. Ai , W. Yan , H. Duan , W. Chen , X. Li , T. Wang , C. Zhang , X. Huang , Nat. Commun. 2018, 9, 5236. 30531797 10.1038/s41467-018-07678-w PMC6286314

[78] Q. Qin , H. Jang , Y. Wang , L. Zhang , Z. Li , M. G. Kim , S. Liu , X. Liu , J. Cho , Adv. Energy Mater. 2021, 11, 2003561.

[79] Y. Zhu , W. Zhou , Z. G. Chen , Y. Chen , C. Su , M. O. Tadé , Z. Shao , Nat. Commun. 2015, 127, 3969.

[80] I. Yamada , H. Fujii , A. Takamatsu , H. Ikeno , K. Wada , H. Tsukasaki , S. Kawaguchi , S. Mori , S. Yagi , Adv. Mater. 2017, 29, 1603004. 10.1002/adma.201603004 27885701

[81] L. Trotochaud , S. L. Young , J. K. Ranney , S. W. Boettcher , J. Am. Chem. Soc. 2014, 136, 6744. 24779732 10.1021/ja502379c

[82] X. Lu , C. Zhao , Nat. Commun. 2015, 6, 6616. 25776015 10.1038/ncomms7616 PMC4382694

[83] J. S. Kanady , E. Y. Tsui , M. W. Day , T. Agapie , Science 2011, 333, 733. 21817047 10.1126/science.1206036

[84] J. J. Concepcion , J. W. Jurss , J. L. Templeton , T. J. Meyer , J. Am. Chem. Soc. 2008, 130, 16462. 19554681 10.1021/ja8059649

[85] J. Lloret Fillol , Z. Codolà Duch , I. Garcia Bosch , L. Gómez Martín , J. J. Pla , M. Costas Salgueiro , Nat. Chem. 2011, 3, 807. 21941254 10.1038/nchem.1140

[86] M. Okamura , M. Kondo , R. Kuga , Y. Kurashige , T. Yanai , S. Hayami , V. K. Praneeth , M. Yoshida , K. Yoneda , S. Kawata , Nature 2016, 530, 465. 26863188 10.1038/nature16529

[87] L. Duan , A. Fischer , Y. Xu , L. Sun , J. Am. Chem. Soc. 2009, 131, 10397. 19601625 10.1021/ja9034686

[88] S. Romain , L. Vigara , A. Llobet , Acc. Chem. Res. 2009, 42, 1944. 19908829 10.1021/ar900240w

[89] L. Duan , F. Bozoglian , S. Mandal , B. Stewart , T. Privalov , A. Llobet , L. Sun , Nat. Chem. 2012, 4, 418. 22522263 10.1038/nchem.1301

[90] S. Neudeck , S. Maji , I. López , S. Meyer , F. Meyer , A. Llobet , J. Am. Chem. Soc. 2014, 136, 24. 24328119 10.1021/ja409974b

[91] L. Duan , C. M. Araujo , M. S. Ahlquist , L. Sun , Proc. Natl. Acad. Sci. USA 2012, 109, 15584. 22753518 10.1073/pnas.1118347109 PMC3465398

[92] B. Yao , Y. Chen , Y. Yan , Y. Yang , H. Xing , Y. Xu , D. Jiao , Z. Xing , D. Wang , X. Yang , Angew. Chem. Int. Ed. 2024, 10.1002/anie.202416141. 39500742

[93] J. Xu , H. Jin , T. Lu , J. Li , Y. Liu , K. Davey , Y. Zheng , S.‐Z. Qiao , Sci. Adv. 2023, 9, eadh1718. 37352343 10.1126/sciadv.adh1718 PMC10289644

[94] H. Jin , X. Liu , P. An , C. Tang , H. Yu , Q. Zhang , H.‐J. Peng , L. Gu , Y. Zheng , T. Song , Nat. Commun. 2023, 14, 354. 36681684 10.1038/s41467-023-35913-6 PMC9867741

[95] F. Liu , D. Zhang , F. She , Z. Yu , L. Lai , H. Li , L. Wei , Y. Chen , ACS Catal. 2024, 14, 9176.

[96] J. Timoshenko , B. Roldan Cuenya , Chem. Rev. 2020, 121, 882. 32986414 10.1021/acs.chemrev.0c00396 PMC7844833

[97] Z. Shi , J. Li , Y. Wang , S. Liu , J. Zhu , J. Yang , X. Wang , J. Ni , Z. Jiang , L. Zhang , Nat. Commun. 2023, 14, 843. 36792586 10.1038/s41467-023-36380-9 PMC9932065

[98] S. Liu , H. Tan , Y. C. Huang , Q. Zhang , H. Lin , L. Li , Z. Hu , W. H. Huang , C. W. Pao , J. F. Lee , Adv. Mater. 2023, 35, 2305659. 10.1002/adma.202305659 37620729

[99] X. Zheng , J. Yang , P. Li , Q. Wang , J. Wu , E. Zhang , S. Chen , Z. Zhuang , W. Lai , S. Dou , Sci. Adv. 2023, 9, eadi8025. 37851800 10.1126/sciadv.adi8025 PMC10584348

[100] T. Zhao , Y. Wang , S. Karuturi , K. Catchpole , Q. Zhang , C. Zhao , Carbon Energy 2020, 2, 582.

[101] S.‐Y. Hong , Z.‐C. Yao , X. Cheng , Z. Jiang , T. Tang , J.‐S. Hu , J. Phys. Chem. C 2024, 128, 17219.

[102] Z. Xu , Z. Liang , W. Guo , R. Zou , Coord. Chem. Rev. 2021, 436, 213824.

[103] A. Vimont , F. Thibault‐Starzyk , M. Daturi , Chem. Soc. Rev. 2010, 39, 4928. 21038035 10.1039/b919543m

[104] S. Wu , M. Taylor , H. Guo , S. Wang , C. Han , J. Vongsvivut , Q. Meyer , Q. Sun , J. Ho , C. Zhao , Angew. Chem., Int. Ed. 2024, 63, 202412455. 10.1002/anie.202412455 39390734

[105] X. Lei , Q. Tang , Y. Zheng , P. Kidkhunthod , X. Zhou , B. Ji , Y. Tang , Nat. Sustainability 2023, 6, 816.

[106] S. W. K. Dastafkan , C. Rong , Q. Meyer , Y. Li , Q. Zhang , C. Zhao , Adv. Funct. Mater. 2022, 32, 2107342.

[107] L. Li , G. Zhang , C. Zhou , F. Lv , Y. Tan , Y. Han , H. Luo , D. Wang , Y. Liu , C. Shang , Nat. Commun. 2024, 15, 4974. 38862507 10.1038/s41467-024-49281-2 PMC11166638

[108] G. Mestl , P. Ruiz , B. Delmon , H. Knozinger , J. Phys. Chem. C 1994, 98, 11269.

[109] J.‐C. Dong , X.‐G. Zhang , V. Briega‐Martos , X. Jin , J. Yang , S. Chen , Z.‐L. Yang , D.‐Y. Wu , J. M. Feliu , C. T. Williams , Nat. Energy 2019, 4, 60.

[110] K. H. Cho , S. Park , H. Seo , S. Choi , M. Y. Lee , C. Ko , K. T. Nam , Angew. Chem., Int. Ed. 2021, 60, 4673. 10.1002/anie.202014551 33417273

[111] S. Lee , L. Bai , X. Hu , Angew. Chem., Int. Ed. 2020, 132, 8149.

[112] S. Lee , K. Banjac , M. Lingenfelder , X. Hu , Angew. Chem., Int. Ed. 2019, 131, 10401. 10.1002/anie.201903200 PMC6771717 31106463

[113] C. Jing , T. Yuan , L. Li , J. Li , Z. Qian , J. Zhou , Y. Wang , S. Xi , N. Zhang , H.‐J. Lin , ACS Catal. 2022, 12, 10276.

[114] Y. Sun , R. Li , X. Chen , J. Wu , Y. Xie , X. Wang , K. Ma , L. Wang , Z. Zhang , Q. Liao , Adv. Energy Mater. 2021, 11, 2003755.

[115] Y. Deng , B. S. Yeo , ACS Catal. 2017, 7, 7873.

[116] O. Diaz‐Morales , F. Calle‐Vallejo , C. de Munck , M. T. Koper , Chem. Sci. 2013, 4, 2334.

[117] S. Pan , H. Li , D. Liu , R. Huang , X. Pan , D. Ren , J. Li , M. Shakouri , Q. Zhang , M. Wang , Nat. Commun. 2022, 13, 2294. 35484271 10.1038/s41467-022-30064-6 PMC9050677

[118] J. Dong , Z. Qian , P. Xu , M.‐F. Yue , R.‐Y. Zhou , Y. Wang , Z.‐A. Nan , S. Huang , Q. Dong , J.‐F. Li , Chem. Sci. 2022, 13, 5639. 35694335 10.1039/d2sc01043g PMC9116351

[119] Y. Wang , Y. Wen , Y. Cheng , X. Chen , M. Zhuansun , T. Wang , J. Li , D. Meira , H. Sun , J. Wei , Nano Res. 2024, 17, 1165.

[120] Y. Wen , P. Chen , L. Wang , S. Li , Z. Wang , J. Abed , X. Mao , Y. Min , C. T. Dinh , P. D. Luna , J. Am. Chem. Soc. 2021, 143, 6482. 33891414 10.1021/jacs.1c00384

[121] Q. Sun , X. Tan , C. Jia , C. Rong , S. Wang , C. Han , Y. Xiao , H. Qi , S. C. Smith , C. Zhao , Adv. Funct. Mater. 2024, 34, 2406281.

[122] Y. Wang , M. Zhang , Z. Kang , L. Shi , Y. Shen , B. Tian , Y. Zou , H. Chen , X. Zou , Nat. Commun. 2023, 14, 5119. 37612274 10.1038/s41467-023-40912-8 PMC10447464

[123] J. Ferreira de Araújo , F. Dionigi , T. Merzdorf , H. S. Oh , P. Strasser , Angew. Chem., Int. Ed. 2021, 60, 14981. 10.1002/anie.202101698 PMC8251801 33830603

[124] M. Görlin , J. Ferreira de Araújo , H. Schmies , D. Bernsmeier , S. r. Dresp , M. Gliech , Z. Jusys , P. Chernev , R. Kraehnert , H. Dau , J. Am. Chem. Soc. 2017, 139, 2070. 28080038 10.1021/jacs.6b12250

[125] Z. Shi , Y. Wang , J. Li , X. Wang , Y. Wang , Y. Li , W. Xu , Z. Jiang , C. Liu , W. Xing , Joule 2021, 5, 2164.

[126] Z. Yang , F. Lai , Q. Mao , C. Liu , S. Peng , X. Liu , T. Zhang , Adv. Mater. 2024, 10.1002/adma.202412950. 39558778

[127] J. Yu , T. Zeng , H. Wang , H. Zhang , Y. Sun , L. Chen , S. Song , L. Li , H. Shi , Chem. Eng. J. 2020, 394, 124458.

[128] I. C. Man , H. Y. Su , F. Calle‐Vallejo , H. A. Hansen , J. I. Martínez , N. G. Inoglu , J. Kitchin , T. F. Jaramillo , J. K. Nørskov , J. Rossmeisl , ChemCatChem 2011, 3, 1159.

[129] Z. Shi , J. Li , J. Jiang , Y. Wang , X. Wang , Y. Li , L. Yang , Y. Chu , J. Bai , J. Yang , Angew. Chem., Int. Ed. 2022, 61, e202212341. 10.1002/anie.202212341 36254795

[130] X. Zhang , C. Feng , B. Dong , C. Liu , Y. Chai , Adv. Mater. 2023, 35, 2207066. 10.1002/adma.202207066 36645873

[131] H. Guo , S. Wu , W. Chen , Z. Su , Q. Wang , N. Sharma , C. Rong , S. Fleischmann , Z. Liu , C. Zhao , Adv. Mater. 2024, 36, 2307118. 10.1002/adma.202307118 38016087

[132] Z. Wu , Q. Li , G. Xu , W. Jin , W. Xiao , Z. Li , T. Ma , S. Feng , L. Wang , Adv. Mater. 2024, 36, 2311018. 10.1002/adma.202311018 38101817

[133] P. Yang , F. Liu , X. Zang , L. Xin , W. Xiao , G. Xu , H. Li , Z. Li , T. Ma , J. Wang , Adv. Energy Mater. 2024, 14, 2303384.

[134] B. Zhou , J. Wang , L. Guo , H. Li , W. Xiao , G. Xu , D. Chen , C. Li , Y. Du , H. Ding , Adv. Energy Mater. 2024, 14, 2402372.

[135] F. Xie , X. Cui , X. Zhi , D. Yao , B. Johannessen , T. Lin , J. Tang , T. B. Woodfield , L. Gu , S.‐Z. Qiao , Nat. Synth. 2023, 2, 129.

[136] J. Lu , Acc. Chem. Res. 2022, 3, 358.

[137] S. Liu , B. Jia , Y. Wang , Y. Zhao , L. Liu , F. Fan , Y. Qin , J. Liu , Y. Jiang , H. Liu , Adv. Mater. 2024, 36, 2409530. 10.1002/adma.202409530 39344144

[138] X. Wang , J. Hu , T. Lu , H. Wang , D. Sun , Y. Tang , H. Li , G. Fu , Angew. Chem. Int. Ed. 2024, 10.1002/anie.202415306.

